# Prospective associations of epigenetic age estimators with Alzheimer's disease atrophy patterns among older women: The Women's Health Initiative Memory Study

**DOI:** 10.1002/alz70856_099085

**Published:** 2025-12-24

**Authors:** Linda K. McEvoy, Bowei Zhang, Steve Nguyen, Ramon Casanova, Ake Lu, Steve Horvath, Mark A. Espeland, Stephen R. Rapp, Adam Maihofer, Caroline Nievergelt, Andrea Z. LaCroix, Susan M. Resnick, Kenneth Beckman, Aladdin H. Shadyab

**Affiliations:** ^1^ Kaiser Permanente Washington Health Research Institute, Seattle, WA, USA; ^2^ University of California San Diego, La Jolla, CA, USA; ^3^ Wake Forest University School of Medicine, Winston‐Salem, NC, USA; ^4^ Altos Labs, Cambridge, Cambridgeshire, United Kingdom; ^5^ Wake Forest University, Winston‐Salem, NC, USA; ^6^ National Institute on Aging, National Institutes of Health, Baltimore, MD, USA; ^7^ University of Minnesota Genomics Center, Minneapolis, MN, USA

## Abstract

**Background:**

Multiple epigenetic age estimators, indicating faster biological aging relative to chronological age, are associated with health outcomes including cardiovascular disease and mortality. Few studies have examined prospective associations of epigenetic age estimators with atrophy in brain regions vulnerable to Alzheimer's disease (AD).

**Methods:**

Women enrolled in the Women's Health Initiative Memory Study (*n* = 1196, mean age=70.0 ±4 years) without prior dementia at baseline (1995‐1998) underwent structural MR imaging on average 8 years later. We calculated a validated measure of neuroanatomic AD risk that captures gray matter tissue atrophy patterns, the AD Pattern Similarity (AD‐PS) score, which has been shown to predict mild cognitive impairment and probable dementia. We examined the association of seven epigenetic age estimators (Horvath age acceleration (AA), Hannum AA, AgeAccelPheno, IEAA, EEAA, AgeAccelGrim2, and DunedinPACE) with the log of AD‐PS score using multiple linear regression adjusting for age, education, race, ethnicity, smoking, hormone therapy trial arm, physical activity, body mass index, comorbidities (diabetes, CVD, cancer), intracranial volume, and blood cell composition. We additionally evaluated effect modification by *APOE ε4* status.

**Results:**

Of the seven epigenetic age estimators, only AgeAccelGrim2 (b=0.0172, 95% CI 0.006 – 0.029; *p* = .003) and EEAA (b=0.0115, 95% CI 0.002 – 0.021; *p* = .02) were associated with the AD‐PS score, with greater age acceleration associated with higher AD‐PS score. Neither of these age estimators interacted with *APOE ε4* status. Of the components of AgeAccelGrim2, only the DNA methylation (DNAm) surrogate of smoking pack years (b=0.023, 95% CI 0.012 – 0.035; *p* <.001) was positively associated with the AD‐PS score. Findings were similar after excluding 46 women with mild cognitive impairment (MCI) or dementia at time of MRI.

**Conclusions:**

AgeAccelGrim2 predicted atrophy in brain regions vulnerable to AD∼8 years later in cognitively healthy women. In a companion study, we found that AgeAccelGrim2 predicted development of MCI/dementia in these women. These findings suggest that this blood‐derived epigenetic measure of accelerated aging may serve as an early biomarker of AD risk and provide further evidence of detrimental associations of smoking with brain health in aging.

